# Supplementary material for: Deletion of wfs1 Impairs Oligodendrocyte Precursor Cells Dorsal Distribution and Myelination Through the wfs1-hmgcs1 Axis in Zebrafish
Source: Biology (Basel). 2026 Aug 16;15(16):1402. doi: 10.3390/biology15161402 (PMC13510151; doi:10.3390/biology15161402)
Supplement: Supplementary file 1 [file biology-15-01402-s001.zip › File S1-Transcriptome sequencing data and differentially expressed genes (DEGs) in wfs1-- zebrafish at 5 dpf/2.KEGG/wfs1KOvsWT/GSEA/wfs1KO/NOTCH_SIGNALING_PATHWAY/NOTCH_SIGNALING_PATHWAY(DRE04330).html]

Details for gene set NOTCH\_SIGNALING\_PATHWAY(DRE04330)[GSEA]

|  || Dataset | deg.sample |
| Phenotype | sample.cls |
| Upregulated in class | wfs1KO |
| GeneSet | NOTCH\_SIGNALING\_PATHWAY(DRE04330) |
| Enrichment Score (ES) | 0.31752464 |
| Normalized Enrichment Score (NES) | 1.2504195 |
| Nominal p-value | 0.168 |
| FDR q-value | 0.32611248 |
| FWER p-Value | 0.981 |
Table: GSEA Results Summary

  

Fig 1: Enrichment plot: NOTCH\_SIGNALING\_PATHWAY(DRE04330)      
 Profile of the Running ES Score & Positions of GeneSet Members on the Rank Ordered List

  

| PROBE | DESCRIPTION (from dataset) | GENE SYMBOL | GENE\_TITLE | RANK IN GENE LIST | RANK METRIC SCORE | RUNNING ES | CORE ENRICHMENT || 1 | 557340\_1 | atxn1a |  |  | 46 | 4.905 | 0.0762 | Yes |
| 2 | 393500\_1 | zgc:66440 |  |  | 206 | 4.159 | 0.1371 | Yes |
| 3 | 566841 | crebbpa |  |  | 530 | 2.904 | 0.1729 | Yes |
| 4 | 497616 | numbl |  |  | 740 | 2.487 | 0.2057 | Yes |
| 5 | 30120\_1 | dlc |  |  | 891 | 2.249 | 0.2366 | Yes |
| 6 | 678649\_1 | zgc:136929 |  |  | 1161 | 1.938 | 0.2588 | Yes |
| 7 | 100148329 | LOC100148329 |  |  | 1538 | 1.633 | 0.2729 | Yes |
| 8 | 793927 | atxn1l |  |  | 1862 | 1.429 | 0.2853 | Yes |
| 9 | 30206 | tle3a |  |  | 3032 | 1.028 | 0.2648 | Yes |
| 10 | 140422 | jag2b |  |  | 3108 | 1.011 | 0.2785 | Yes |
| 11 | 503870 | rbpjl |  |  | 3176 | 0.998 | 0.2922 | Yes |
| 12 | 335134\_1 | heyl |  |  | 3291 | 0.975 | 0.3040 | Yes |
| 13 | 140421 | jag1a |  |  | 3440 | 0.943 | 0.3143 | Yes |
| 14 | 100329328 | zmp:0000001236 |  |  | 3778 | 0.872 | 0.3175 | Yes |
| 15 | 564019 | si:dkey-42p8.3 |  |  | 4418 | 0.757 | 0.3094 | No |
| 16 | 359836 | her15.1 |  |  | 4974 | 0.685 | 0.3028 | No |
| 17 | 402914 | her12 |  |  | 5788 | 0.608 | 0.2869 | No |
| 18 | 568626\_1 | dtx2 |  |  | 5833 | 0.604 | 0.2951 | No |
| 19 | 560657 | adam17b |  |  | 6113 | 0.575 | 0.2954 | No |
| 20 | 559273\_1 | ep300a |  |  | 6707 | 0.519 | 0.2850 | No |
| 21 | 101885597 | LOC101885597 |  |  | 7068 | 0.486 | 0.2813 | No |
| 22 | 100144568 | si:dkey-3h3.3 |  |  | 7113 | 0.481 | 0.2876 | No |
| 23 | 101883874 | LOC101883874 |  |  | 8802 | 0.381 | 0.2405 | No |
| 24 | 567335 | maml1 |  |  | 8891 | 0.375 | 0.2437 | No |
| 25 | 493633 | mfng |  |  | 9022 | 0.366 | 0.2454 | No |
| 26 | 566455 | jag2a |  |  | 10351 | 0.282 | 0.2081 | No |
| 27 | 140613\_1 | her9 |  |  | 10832 | 0.253 | 0.1970 | No |
| 28 | 58026 | psen2 |  |  | 11048 | 0.241 | 0.1940 | No |
| 29 | 58065 | notch2 |  |  | 11107 | 0.239 | 0.1960 | No |
| 30 | 494449 | ncstn |  |  | 11363 | 0.224 | 0.1915 | No |
| 31 | 30221 | psen1 |  |  | 11456 | 0.219 | 0.1921 | No |
| 32 | 563920 | dll4 |  |  | 11461 | 0.219 | 0.1954 | No |
| 33 | 101886283 | maml3 |  |  | 11630 | 0.209 | 0.1935 | No |
| 34 | 30300 | her2 |  |  | 11791 | 0.201 | 0.1916 | No |
| 35 | 565841 | atxn1b |  |  | 12504 | 0.164 | 0.1718 | No |
| 36 | 30131 | dla |  |  | 12964 | 0.142 | 0.1596 | No |
| 37 | 569863 | dvl1a |  |  | 13418 | 0.120 | 0.1473 | No |
| 38 | 567111 | crebbpb |  |  | 13470 | 0.117 | 0.1475 | No |
| 39 | 30158 | lfng |  |  | 14026 | 0.091 | 0.1315 | No |
| 40 | 100007463 | tle3b |  |  | 14593 | 0.065 | 0.1148 | No |
| 41 | 100149066 | her4.4 |  |  | 15089 | 0.042 | 0.0999 | No |
| 42 | 571706 | zmp:0000000926 |  |  | 15153 | 0.039 | 0.0985 | No |
| 43 | 100534909 | LOC100534909 |  |  | 15545 | 0.020 | 0.0865 | No |
| 44 | 565612 | ep300b |  |  | 15569 | 0.019 | 0.0861 | No |
| 45 | 65229\_1 | ctbp1 |  |  | 15700 | 0.012 | 0.0822 | No |
| 46 | 58066 | notch3 |  |  | 15784 | 0.007 | 0.0797 | No |
| 47 | 30718 | notch1a |  |  | 15928 | 0.001 | 0.0752 | No |
| 48 | 80972 | dvl3a |  |  | 16463 | -0.016 | 0.0587 | No |
| 49 | 324142 | adam17a |  |  | 16639 | -0.021 | 0.0535 | No |
| 50 | 559273 | ep300a |  |  | 16774 | -0.025 | 0.0497 | No |
| 51 | 386772 | rbpja |  |  | 16857 | -0.029 | 0.0476 | No |
| 52 | 30141 | dlb |  |  | 17467 | -0.052 | 0.0292 | No |
| 53 | 560032 | dvl1b |  |  | 17603 | -0.058 | 0.0259 | No |
| 54 | 561503 | ncor2 |  |  | 18023 | -0.077 | 0.0140 | No |
| 55 | 559336 | zgc:114200 |  |  | 18205 | -0.085 | 0.0096 | No |
| 56 | 563306 | rbpjb |  |  | 18815 | -0.114 | -0.0077 | No |
| 57 | 140423 | jag1b |  |  | 18851 | -0.116 | -0.0070 | No |
| 58 | 192302 | hdac1 |  |  | 19034 | -0.123 | -0.0108 | No |
| 59 | 559273\_2 | ep300a |  |  | 19072 | -0.125 | -0.0100 | No |
| 60 | 323630 | dvl2 |  |  | 19187 | -0.130 | -0.0115 | No |
| 61 | 30138 | dld |  |  | 20320 | -0.180 | -0.0442 | No |
| 62 | 565013 | tle2 |  |  | 20348 | -0.181 | -0.0422 | No |
| 63 | 65230 | ctbp2a |  |  | 20504 | -0.188 | -0.0441 | No |
| 64 | 794892 | notch1b |  |  | 20626 | -0.195 | -0.0448 | No |
| 65 | 567111\_1 | crebbpb |  |  | 20683 | -0.198 | -0.0435 | No |
| 66 | 414848 | rfng |  |  | 20978 | -0.214 | -0.0493 | No |
| 67 | 548605 | ctbp2l |  |  | 21883 | -0.261 | -0.0736 | No |
| 68 | 386808\_1 | aph1b |  |  | 22275 | -0.282 | -0.0814 | No |
| 69 | 101886356 | LOC101886356 |  |  | 22442 | -0.292 | -0.0820 | No |
| 70 | 30288\_1 | her6 |  |  | 23038 | -0.330 | -0.0955 | No |
| 71 | 563942 | kat2b |  |  | 23122 | -0.336 | -0.0928 | No |
| 72 | 334026 | si:dkey-40c23.2 |  |  | 23166 | -0.338 | -0.0888 | No |
| 73 | 402810 | psenen |  |  | 23567 | -0.365 | -0.0956 | No |
| 74 | 548605\_1 | ctbp2l |  |  | 23705 | -0.373 | -0.0940 | No |
| 75 | 570481 | si:ch211-119e14.2 |  |  | 23870 | -0.384 | -0.0931 | No |
| 76 | 553421 | si:ch73-167c12.2 |  |  | 24204 | -0.407 | -0.0971 | No |
| 77 | 565013\_1 | tle2 |  |  | 24234 | -0.409 | -0.0915 | No |
| 78 | 321852 | snw1 |  |  | 25021 | -0.447 | -0.1092 | No |
| 79 | 555517 | kat2a |  |  | 25644 | -0.498 | -0.1209 | No |
| 80 | 100149082 | dvl3 |  |  | 26821 | -0.611 | -0.1482 | No |
| 81 | 58146 | hey2 |  |  | 27615 | -0.689 | -0.1622 | No |
| 82 | 497616\_1 | numbl |  |  | 27621 | -0.690 | -0.1514 | No |
| 83 | 30120 | dlc |  |  | 28065 | -0.751 | -0.1535 | No |
| 84 | 65229 | ctbp1 |  |  | 28678 | -0.852 | -0.1592 | No |
| 85 | 30206\_1 | tle3a |  |  | 28710 | -0.860 | -0.1466 | No |
| 86 | 692064 | numb |  |  | 28817 | -0.882 | -0.1360 | No |
| 87 | 58008 | hey1 |  |  | 28941 | -0.907 | -0.1255 | No |
| 88 | 100037355 | dtx4 |  |  | 29260 | -0.992 | -0.1198 | No |
| 89 | 140422\_1 | jag2b |  |  | 30471 | -1.488 | -0.1343 | No |
| 90 | 566841\_1 | crebbpa |  |  | 30571 | -1.563 | -0.1126 | No |
| 91 | 557340 | atxn1a |  |  | 31122 | -2.114 | -0.0964 | No |
| 92 | 678649 | zgc:136929 |  |  | 31464 | -2.814 | -0.0626 | No |
| 93 | 393500 | zgc:66440 |  |  | 31826 | -4.799 | 0.0020 | No |
Table: GSEA details [plain text format]

  

Fig 2: NOTCH\_SIGNALING\_PATHWAY(DRE04330)      
 Blue-Pink O' Gram in the Space of the Analyzed GeneSet

  

Fig 3: NOTCH\_SIGNALING\_PATHWAY(DRE04330): Random ES distribution      
 Gene set null distribution of ES for **NOTCH\_SIGNALING\_PATHWAY(DRE04330)**
